# Supplementary material for: PCID2 dysregulates transcription and viral RNA processing to promote HIV-1 latency
Source: iScience. 2024 Feb 6;27(3):109152. doi: 10.1016/j.isci.2024.109152 (PMC10879814; doi:10.1016/j.isci.2024.109152)
Supplement: Document S1. Figures S1–S8 and Table S2 [file mmc1.pdf]

iScience, Volume *27*

Supplemental information

PCID2 dysregulates transcription and viral RNA processing to promote HIV-1 latency

Raquel Crespo, Enrico Ne, Julian Reinders, Jenny I.J. Meier, Chengcheng Li, Sanne Jansen, Alicja Górska, Selin Koçer, Tsung Wai Kan, Wouter Doff, Dick Dekkers, Jeroen Demmers, Robert-Jan Palstra, Shringar Rao, and Tokameh Mahmoudi

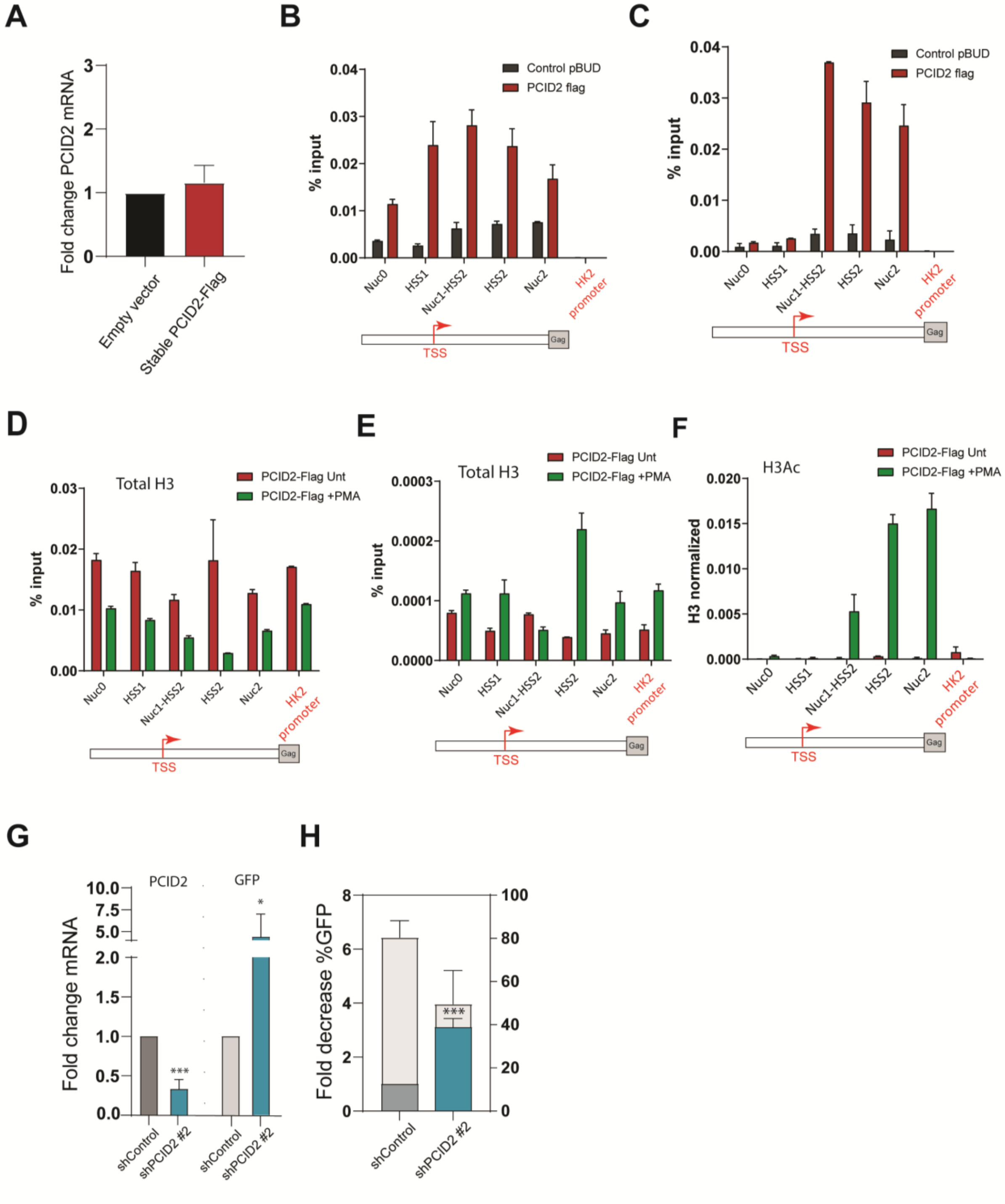

A
Fold change PCID2 mRNA
Empty vector
Stable PCID2-Flag
B
% input
Control pBUD
PCID2 flag
Nuc0
HSS1
Nuc1-HSS2
HSS2
Nuc2
HK2 promoter
TSS
Gag
C
% input
Control pBUD
PCID2 flag
Nuc0
HSS1
Nuc1-HSS2
HSS2
Nuc2
HK2 promoter
TSS
Gag
D
Total H3
% input
PCID2-Flag Unt
PCID2-Flag +PMA
Nuc0
HSS1
Nuc1-HSS2
HSS2
Nuc2
HK2 promoter
TSS
Gag
E
Total H3
% input
PCID2-Flag Unt
PCID2-Flag +PMA
Nuc0
HSS1
Nuc1-HSS2
HSS2
Nuc2
HK2 promoter
TSS
Gag
F
H3Ac
H3 normalized
PCID2-Flag Unt
PCID2-Flag +PMA
Nuc0
HSS1
Nuc1-HSS2
HSS2
Nuc2
HK2 promoter
TSS
Gag
G
PCID2
GFP
Fold change mRNA
***
*
shControl
shPCID2 #2
shControl
shPCID2 #2
H
Fold decrease %GFP
***
shControl
shPCID2 #2

Figure S1. ChIP-qPCR analysis of PCID2-Flag enrichment at the HIV-1 LTR. Related to Figure 1

A. Gene expression analysis of exogenously expressing PCID2-Flag J-Lat 11.1 cells (Stable PCID2-Flag) relative to Control lines (empty vector) and normalized to cyclophilin A. Bars represent mean of 3 separate collections and error lines represent SEM. **B-C**. Enrichment of PCID2-Flag at the HIV-1 LTR expressed as % of input in control and PCID2-Flag expressing J-Lat 11.1 lines as assessed by chromatin immunoprecipitation (ChIP) coupled with qPCR. Primers spanning across the HIV-1 promoter were used to assess relative enrichment of PCID2-Flag at sequential regions of the LTR. HK2 promoter was used as a control genomic region. ChIP-qPCR corresponds to two independent chromatin preparations (**B** and **C**), bars and error lines represent, respectively, mean and SEM of technical duplos. **D.** ChIP-qPCR analysis of total Histone 3 enrichment at the HIV-1 promoter in untreated and PMA treated PCID2-Flag expressing J-Lat 11.1 lines. Total H3 presence at the HIV-1 LTR is represented as % input and is used for normalization of acetylated Histone 3 as presented in Figure 1D. ChIP-qPCR corresponds to one chromatin preparation, bars and error lines represent, respectively, mean and SD of technical duplos. **E-F.** ChIP-qPCR analysis of total Histone 3 (E) and acetylated Histone 3 (F) enrichment at the HIV-1 promoter in untreated and PMA treated PCID2-Flag expressing J-Lat 11.1 lines. Total H3 presence at the HIV-1 LTR is represented as % input. H3Ac enrichment at the HIV-1 LTR is represented as relative enrichment normalized to Total H3 as shown in D. ChIP-qPCR corresponds to one chromatin preparation, bars and error lines represent, respectively, mean and SD of technical duplos. **G.** Gene expression analysis of shRNA-mediated knockdown of PCID2 and GFP mRNA fold induction in shPCID2 cells relative to shControl and normalized to cyclophilin A. Bars represent mean of 5 independent shRNA-mediated knockdown experiments and error lines represent SEM (n=5). Statistical significance was determined by t-test; *p<0.05, ***p<0.001. **H.** Fold increase in the % of GFP (left y axes) and viability (right y axes) in shControl or shPCID2 J-Lat 11.1 cells as measured by flow cytometry. Bars represent mean of 5 independent shRNA-mediated knockdown experiments and error lines represent SEM. Statistical significance was determined by t-test; ***p<0.001. Raw values for percentage of GFP are available in Supplementary Table 2.

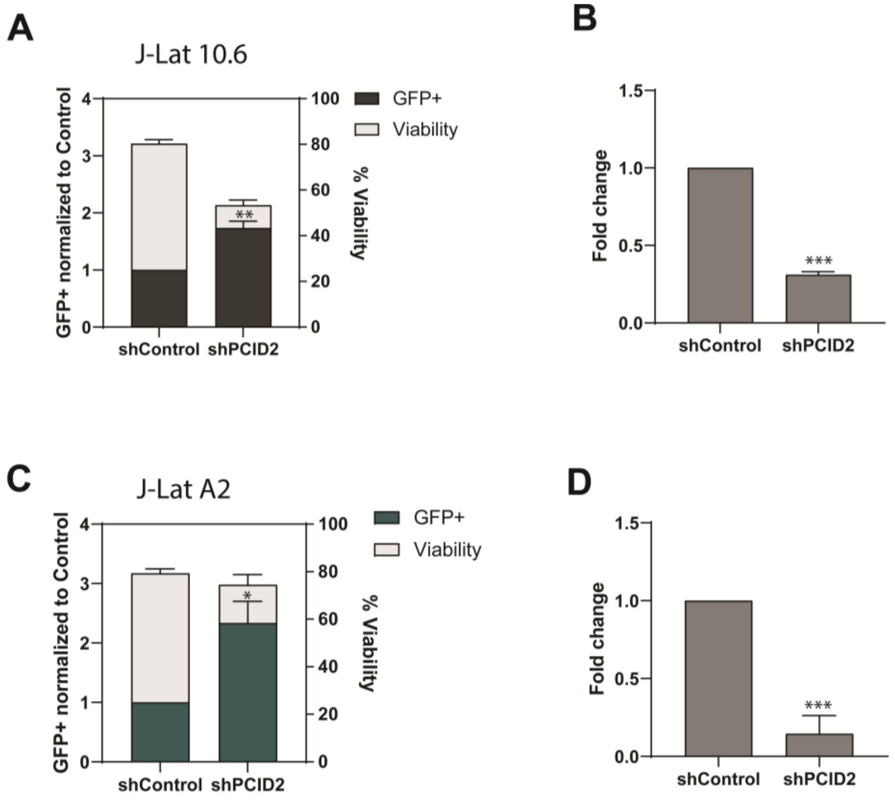

Figure S2. PCID2 knockdown in J-Lat clones 10.6 and A2. Related to Figure 1

A. Fold increase in the % of GFP (left y axes) and viability (right y axes) in shControl or shPCID2 J-Lat 10.6 cells as measured by flow cytometry. Bars represent mean of 3 independent shRNA-mediated knockdown experiments and error lines represent SEM. Statistical significance was determined by t-test; **$p<0.01$. Raw values for percentage of GFP are available in Supplementary Table 2. **B.** Gene expression analysis of shRNA-mediated knockdown of PCID2 in J-Lat 10.6 cells relative to shControl and normalized to cyclophilin A. Bars represent mean of 3 independent shRNA-mediated knockdown experiments and error lines represent SEM. Statistical significance was determined by t-test; ***$p<0.001$. **C.** Fold increase in the % of GFP (left y axes) and viability (right y axes) in shControl or shPCID2 J-Lat A2 cells as measured by flow cytometry. Bars represent mean of 3 independent shRNA-mediated knockdown experiments and error lines represent SEM. Statistical significance was determined by t-test; *$p<0.05$. Raw values for percentage of GFP are available in Supplementary Table 2. **D.** Gene expression analysis of shRNA-mediated knockdown of PCID2 in J-Lat A2 cells relative to shControl and normalized to cyclophilin A. Bars represent mean of 3 independent shRNA-mediated knockdown experiments and error lines represent SEM. Statistical significance was determined by t-test; ***$p<0.001$.

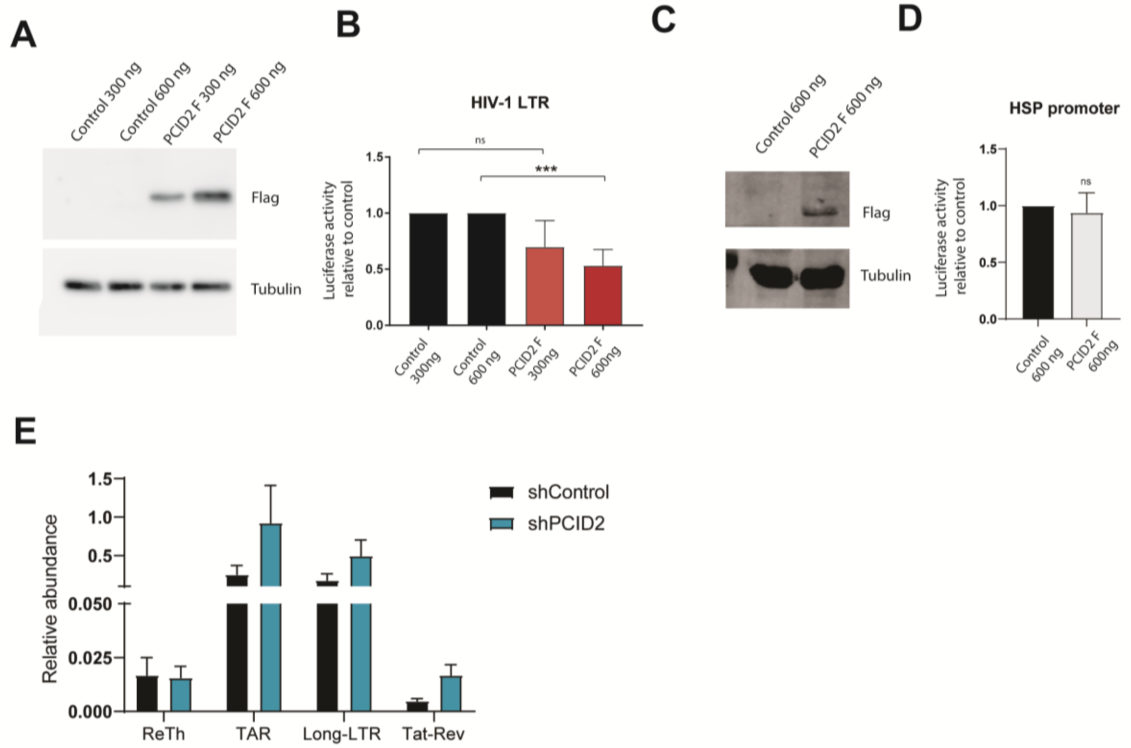

Figure S3. Effect of PCID2 overexpression on HIV-1 gene expression. Related to Figure 2 and 3

A. Western blot analysis of PCID2-Flag in transiently co-nucleofected Jurkat cells with pNL4.3.Luc.R-E-, pRL *Renilla* and either pBud-Control or pBud-PCID2-Flag at 300 or 600 ng. Cells were collected 48 hours post-nucleofection. Beta tubulin was used as a loading control. **B.** Change in HIV-1 gene expression in Jurkat cells transiently expressing PCID2-Flag was assessed by luciferase assay. Jurkat cells were co-nucleofected pNL4.3.Luc.R-E- , pRL *Renilla* and pBud-Control or pBud-PCID2-Flag at 300 or 600 ng. Firefly luciferase units were normalized to Renilla luciferase units and relative change in luciferase activity was normalized to control. Bars represent mean of 3 independent experiments and error lines represent SEM. Statistical significance was determined by t-test; ***$p<0.001$. **C.** Western blot analysis of PCID2-Flag in transiently co-nucleofected Jurkat cells with pGL3-HSP vector, pRL *Renilla* and either pBud-Control or pBud-PCID2-Flag at 600 ng. Cells were collected 48 hours post-nucleofection. Beta tubulin was used as a loading control. **D.** Change in HIV-1 gene expression in Jurkat cells transiently expressing PCID2-Flag was assessed by luciferase assay. Jurkat cells were co-nucleofected pGL3-HSP vector, pRL *Renilla* and pBud-Control or pBud-PCID2-Flag at 600 ng. Firefly luciferase units were normalized to Renilla luciferase units and relative change in luciferase activity was normalized to control. Bars represent mean of 3 independent experiments and error lines represent SEM. Statistical significance was determined by t-test; ns= not significant. **E.** Relative abundance of HIV-1 RNA amplicons in shControl and shPCID2 J-Lat 11.1 cells.

A

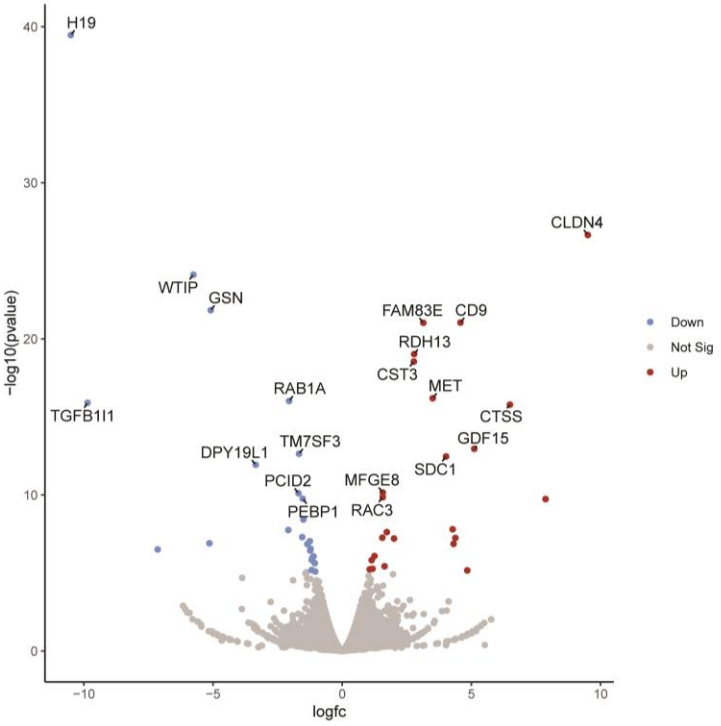

Figure S4. Volcano plot of differentially expressed genes in shPCID2 compared to shControl J-Lat 11.1 cells. Related to Figure 2

A. Volcano plot representation of RNASeq data analysed as differentially expressed genes in shPCID2 compared to shControl J-Lat 11.1 cells.

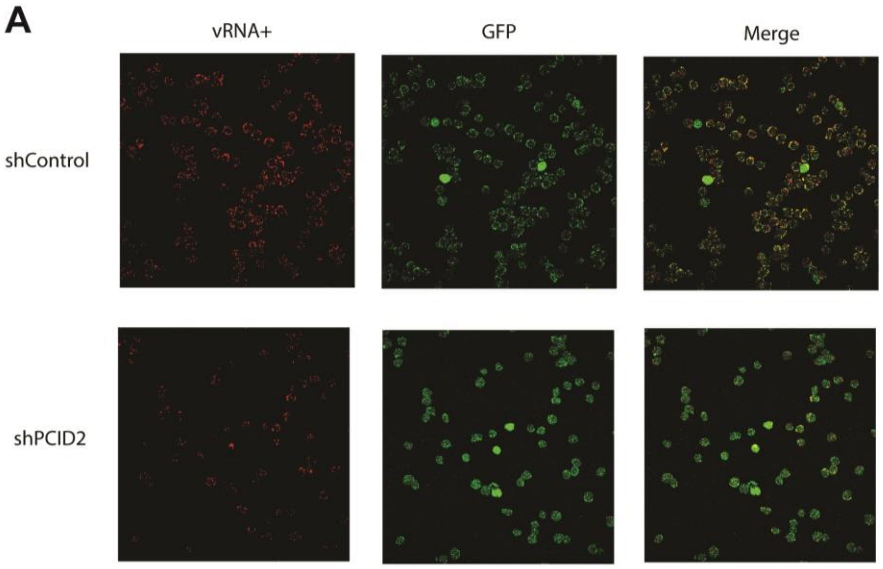

Figure S5. FISH-Flow analysis of shControl and shPCID2 J-Lat 11.1 cells. Related to Figure 3

A. Representative confocal microscopy images from shControl and shPCID2 J-Lat 11.1 cells seeded as a whole population after FISH-Flow analysis as shown in Figure 5C-E.

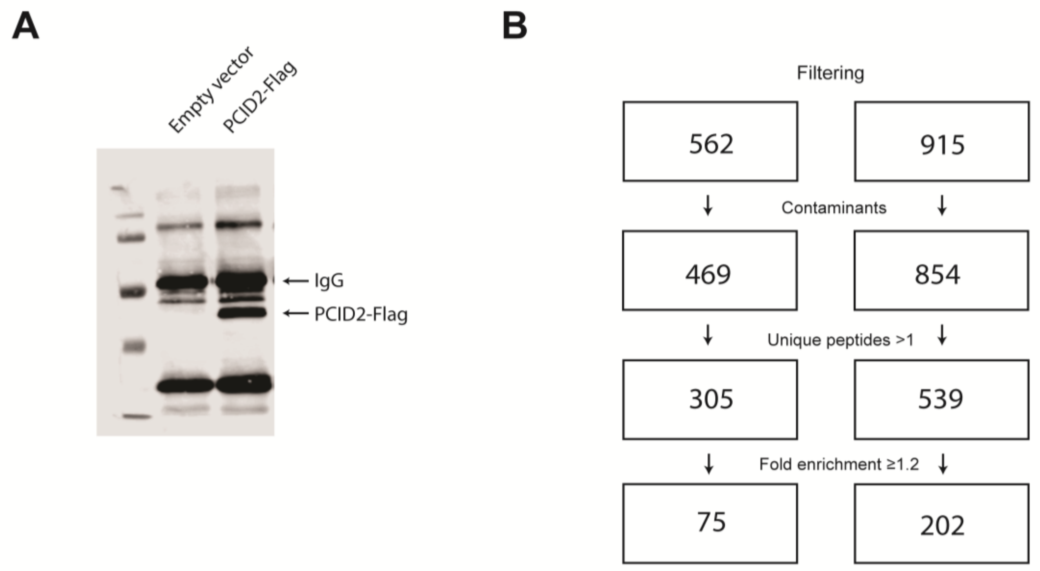

Figure S6. Mass spectrometry hit list filtering flowchart. Related to Figure 4

A. IP-western blot of PCID2-Flag in control and stably PCID2-Flag expressing J-Lat 11.1 samples for subsequent mass spectrometry analysis. **B.** Filtering scheme used for the hits identified in the two IP-MS runs (left and right columns). Numbers represent total amount of hits after each filtering step. First quadrant shows total hits identified. Total amount of hits were filtered out for common contaminants, including keratin and immunoglobulin proteins. Next, we filtered for unique peptides >1 followed by a fold-enrichment calculation in iBAQ values with a 1.2 fold enrichment cut-off.

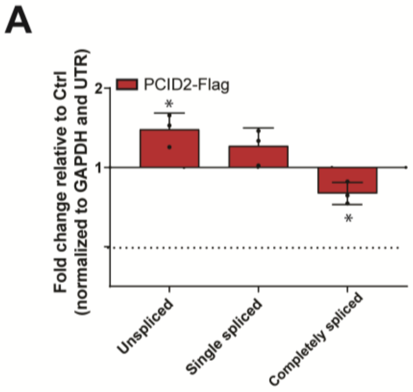

Figure S7. Effect of PCID2 overexpression on HIV-1 alternative splicing. Related to Figure 5

A. Fold change in HIV-1 RNA splicing variants upon PCID2 overexpression in Jurkat cells co-transfected with pNL4.3.Luc.R-E- and pBud-Control or pBud-PCID2-Flag. Values were normalized to GAPDH and UTR HIV-1 transcript. Bars represent mean and error lines represent SEM. Statistical significance was determined by t-test; *p<0.05, **p<0.01.

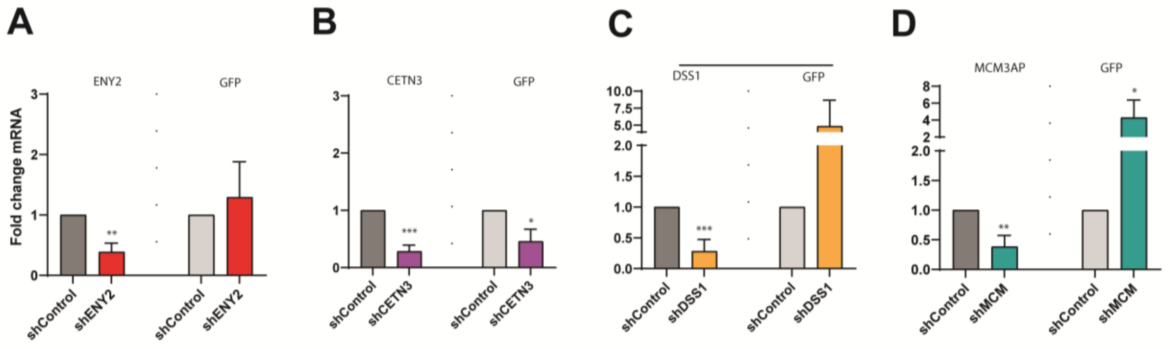

Figure S8. Efficiency of shRNA-mediated knockdown for TREX2 subunits. Related to Figure 6

A. Gene expression analysis of shRNA-mediated knockdown and GFP mRNA fold induction in shENY2 (A), shCETN3 (B), shDSS1 (C), shMCM3AP (D) cells relative to shControl and normalized to cyclophilin A. Bars represent mean of three independent shRNA-mediated knockdown experiments and error lines represent SEM. Statistical significance was determined by t-test; *p<0.05, **p<0.01, ***p<0.001.

Table S2. Values for averaged percentage of GFP across samples used for fold-change calculations in the indicated figures. Related to Figure 1, 6 and Figure S1

**Figure 1G**	**Sample**	**Average % GFP**	**Standard error**
	shControl	1.52	0.79
	shPCID2 #1	4.41	3.49
**Figure 1H**	**Sample**	**Average % GFP**	**Standard error**
	Empty vector	2.905	0.259855729
	PCID2-Flag	0.86	0.125698051
**Figure 1I**	**Sample**	**Average % GFP**	**Standard error**
	Ctrl Untreated	0.56	0.02
	PCID2-Flag Untreated	0.77	0.13
	Ctrl VPA 2mm	7.00	0.76
	PCID2-Flag VPA 2mm	5.53	2.01
	Ctrl Untreated	0.87	0.10
	PCID2-Flag Untreated	1.05	0.10
	Ctrl Pros 200nm	6.26	0.42
	PCID2-Flag Pros 200nm	5.86	0.02
	Ctrl Untreated	0.70	0.21
	PCID2-Flag Untreated	0.79	0.15
	Ctrl K98 10µm	2.26	0.60
	PCID2-Flag K98 10µm	1.59	0.57
**Figure 6D**	**Sample**	**Average % GFP**	**Standard error**
	shControl	1.15	0.94
	shDSS1	3.29	1.81
	shControl	0.64	1.24
	shMCM3AP	0.26	0.27
	shControl	2.09	1.36
	shENY2	2.34	1.51
	shControl	1.35	0.20
	shCETN3	1.27	0.20
**Figure S1H**	**Sample**	**Average % GFP**	**Standard error**
	shControl	1.28	0.17
	shPCID2 #2	3.97	0.85
